# Nuclear Translocation of Soybean MPK6, GmMPK6, Is Mediated by Hydrogen Peroxide in Salt Stress

**DOI:** 10.3390/plants10122611

**Published:** 2021-11-28

**Authors:** Jong Hee Im, Seungmin Son, Jae-Heung Ko, Kyung-Hwan Kim, Chung Sun An, Kyung-Hwan Han

**Affiliations:** 1 School of Biological Sciences, College of Natural Sciences, Seoul National University, Seoul 151-747, Korea; jhim@msu.edu (J.H.I.); linewind@korea.kr (S.S.); ancs@snu.ac.kr (C.S.A.); 2 Department of Horticulture, Michigan State University, East Lansing, MI 48824, USA; 3 National Institute of Agricultural Sciences, Rural Development Administration, Jeonju 54874, Korea; biopiakim@korea.kr; 4 Department of Plant & Environmental New Resources, College of Life Science and Graduate School of Biotechnology, Kyung Hee University, Yongin-si 17104, Korea; jhko@khu.ac.kr; 5 Department of Forestry, Michigan State University, East Lansing, MI 48824, USA

**Keywords:** mitogen-activated protein kinase (MPK), nuclear translocation of MPK, salt stress, soybean, hydrogen peroxide

## Abstract

The plant mitogen-activated protein kinase (MPK) cascade, a highly conserved signal transduction system in eukaryotes, plays a crucial role in the plant’s response to environmental stimuli and phytohormones. It is well-known that nuclear translocation of MPKs is necessary for their activities in mammalian cells. However, the mechanism underlying nuclear translocation of plant MPKs is not well elucidated. In the previous study, it has been shown that soybean MPK6 (GmMPK6) is activated by phosphatidic acid (PA) and hydrogen peroxide (H_2_O_2_), which are two signaling molecules generated during salt stress. Using the two signaling molecules, we investigated how salt stress triggers its translocation to the nucleus. Our results show that the translocation of GmMPK6 to the nucleus is mediated by H_2_O_2_, but not by PA. Furthermore, the translocation was interrupted by diphenylene iodonium (DPI) (an inhibitor of RBOH), confirming that H_2_O_2_ is the signaling molecule for the nuclear translocation of GmMPK6 during salt stress.

## 1. Introduction

High concentration of salt generates osmotic stress and ionic toxicity as well as oxidative damage to the plant. In response to the stress, the plants activate defense responses, such as detoxification, re-established homeostasis of ion, and growth regulation [1]. Various plant mitogen-activated protein kinases (MPKs) are known to be activated by abiotic stress, including AtMPK3, AtMPK4 and AtMPK6 in *Arabidopsis* [2,3,4], OsMPK4 in rice [5], SIMK in alfalfa [6], and GmMPK6 (GMK1) in soybean [7].

During salt stress, two signaling molecules, phosphatidic acid (PA) and hydrogen peroxide (H_2_O_2_), are generated in plants [3,8,9,10]. PA regulates plant growth and stress responses, and induces MPK activity [11,12,13]. The signaling lipid PA is generated when phospholipase D (PLD) hydrolyses structural phospholipids phosphatidylcoline (PC) [14]. Primary short-chain alcohols (e.g., ethanol, *n*-butanol) can substitute for and compete with water during PA production by PLD, generating transphosphatidylalcohols. As such, *n*-butanol can inhibit PA generation [15,16]. H_2_O_2_ is also a well-known signal molecule with its stability and long life time in plant [17]. Nicotinamide adenine dinucleotide phosphate-oxidase (NADPH-oxidase) is localized in the plasma membrane and produces superoxide from electron transfer [18], and the molecule is then changed by dismutation to H_2_O_2_. Activity of NADPH-oxidase is blocked by diphenylene iodonium (DPI) which reduces reactive oxygene species (ROS) generation in mammalian as well as in plant [19,20,21]. In soybean, DPI also reduces ROS generation in salt stress [7].

The MPK signaling pathway transfers signals from the cell membrane to the nucleus and regulates the corresponding gene expression. This transmission of the signals to the nucleus requires the physical translocation of components of the MPK cascade into the nucleus [22]. However, it has not been extensively studied how the nuclear translocation of plant MPKs is modulated. In this report, we show that the nuclear translocation of GmMPK6 is mediated by H_2_O_2_ under salt stress conditions.

## 2. Results

**Figure 1:**
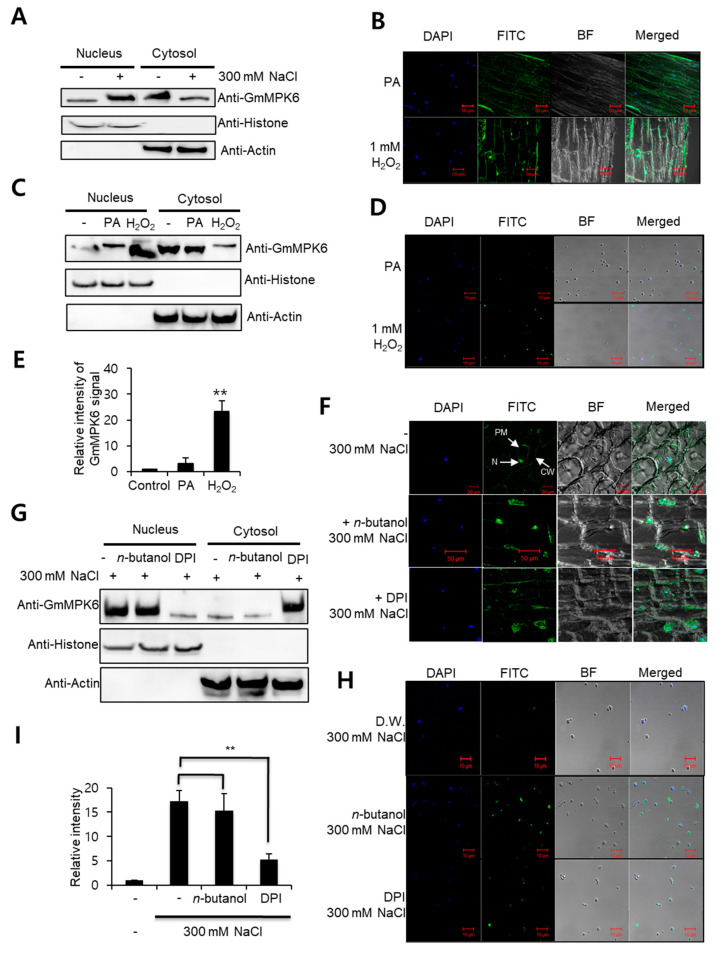
Hydrogen peroxide mediates nuclear translocation of GmMPK6. (**A**) Protein blot analysis of GmMPK6, showing salt treatment increases its protein level in the nucleus. (**B**) Immunolocalization of GmMPK6. Soybean seedlings were treated with 50 μM PA and 1 mM H_2_O_2_ for 60 min, respectively, and longitudinally sectioned. The immunolocalization assay was carried out with anti-GmMPK6 and FITC conjugated secondary antibody. The images were obtained by confocal microscopy (LSM-510, Carl Zeiss, Oberkochen, Germany). (**C**) Protein blot analysis of GmMPK6 in nuclear fraction, showing H_2_O_2_ treatment results in nuclear translocation of GmMPK6. The protein blot analysis was carried out with anti-GmMPK6 antibody. Anti-Histone was used as nucleus fractions marker and anti-Actin as cytosol fractions marker. (**D**) Immunodetection of GmMPK6 in the nuclei. The immunodetection assay was carried out with the nuclei isolated from samples (**B**) using anti-GmMPK6 antibody and FITC conjugated secondary antibody. The images were obtained by confocal microscopy. (**E**) Signal intensity of (**D**). The signal intensity was measured with image J program. Values are means ± SE of three repeats. ** *p* < 0.001. (**F**) Immunolocalization assay of GmMPK6. The soybean seedlings were treated with 1% *n*-butanol or 50 μM DPI for 60 min, followed by 300 mM NaCl treatment for 60 min. Immunolocalization assay with anti-GmMPK6 and FITC conjugated secondary antibody. The images were obtained by confocal microscopy. N: nucleus; PM: plasma membrane; CW: cell wall. (**G**) Protein blot analysis of GmMPK6 in the nuclear fraction of 300 mM NaCl-treated soybean seedlings with or without *n*-butanol or DPI pre-treatment. The protein blot analysis was carried out with anti-GmMPK6 antibody. Anti-Histone was used as nucleus fractions marker and anti-Actin as cytosol fractions marker. (**H**) Immunodetection of GmMPK6 in the nuclei. The immunodetection assay was carried out with the nuclei isolated from samples (**F**) using anti-GmMPK6 antibody and FITC conjugated secondary antibody. The images were obtained by confocal microscopy. (**I**) Signal intensity of (H). The signal intensity was measured with Image J program. Values are means ± SE of three repeats: ** *p* < 0.001.

It has been shown that a soybean MPK, GmMPK6, is activated by PA and/or H_2_O_2_ during salt stress [7,23]. Based on this observation and our data (Figure 1A), we hypothesized that PA and/or H_2_O_2_ may be the nuclear translocation regulators of GmMPK6 under salt stress condition. To investigate whether the nuclear translocation of GmMPK6 is mediated by these two molecules, we performed immunolocalization assays with anti-GmMPK6 antibodies using 7-day-old soybean seedlings treated with PA or H_2_O_2_ for 60 min. GmMPK6 was barely matched with nucleus signals (DAPI) in the PA treatment, but the signal was strongly concentrated and corresponded with DAPI signals in the H_2_O_2_ treatment (Figure 1B), suggesting that the nuclear translocation was induced by the H_2_O_2_ treatment. To further confirm this finding, we carried out protein blot analyses using isolated nuclei. In normal conditions, a large amount of GmMPK6 was detected in the cytosol. This pattern of cytosol localization was also observed with the PA treatment. However, the H_2_O_2_ treatment strongly increased GmMPK6 in the nucleus fraction (Figure 1C). Furthermore, we used isolated nuclei from the samples illustrated in Figure 1B for the analysis of the GmMPK6 signal in the nuclei with immunodetection. While the GmMPK6 signal was not detected in the PA treated nuclei, a strong signal was detected in the H_2_O_2_ treated nuclei (Figure 1D,E), indicating that the nuclear translocation of GmMPK6 is regulated by H_2_O_2_ treatment.

To confirm that the nuclear translocation of GmMPK6 is mediated by ‘endogenous’ H_2_O_2_ during salt stress, we used a pharmacological approach. First, in our effort to reduce PA generation by salt stress, we treated the soybean seedlings with *n*-butanol, a PLD inhibitor, for 60 min, followed by 300 mM NaCl treatment for 60 min. To reduce ROS generation in the soybean seedlings, DPI (inhibitor of H_2_O_2_ generation) was pre-treated to the seedlings for 60 min, followed by 300 mM NaCl treatment for 60 min.

With salt stress treatment, the cytosols of the cells were shrunken, but the GmMPK6 was clearly detected in the nucleus (Figure 1F). This nuclear translocation was not changed by the *n*-butanol pre-treatment, but the GmMPK6 signal was hardly correlated with DAPI signal in the DPI pre-treatment (Figure 1F). To further confirm this nuclear localization of GmMPK6, we carried out protein blot analyses using isolated nuclei and cytosols. The analyses showed strong presence of GmMPK6 proteins in the isolated nuclei when the seedlings were treated with 300 mM NaCl or with *n*-butanol pre-treatment (Figure 1G). However, the GmMPK6 band was diminished by the DPI pre-treatment (Figure 1G). This finding was further confirmed by an immunodetection assay using the isolated nucleus samples (Figure 1H,I). While the GmMPK6 signal was strongly detected in the *n*-butanol pre-treated samples, the signal was clearly reduced in the DPI pre-treatment. Taken together, we conclude that the nuclear translocation of GmMPK6 is mediated by endogenous H_2_O_2_ during salt stress.

## 3. Discussion

As a sessile organism, plants must coordinate their growth and development with their surrounding environmental factors. The mitogen-activated protein kinase (MPK) cascade is a highly conserved signal transduction module involved in transducing extracellular signals (e.g., positional and environmental information) to the nucleus for appropriate biochemical and physiological cellular responses. Of the MPKs, MPK6 is post-translationally activated by salt stress [3,8] and regulates stress adaptation processes [24]. Its translocation to the nucleus is a prerequisite for the MPK to be functional. However, it remains to be elucidated by which mechanism the nuclear translocation is mediated. Using a soybean MPK, we showed that salt stress-induced H_2_O_2_ triggers the translocation of GmMPK6 under salt stress conditions.

It has been shown that exogenous H_2_O_2_ mediates nuclear translocation of MPKs in other plants such as peanut (AhMPK3) and rice (OsBWMK1) [25,26], in support of our findings. In the current study, we used DPI pre-treatment (i.e., inhibitor of RBOH) to clearly show that endogenous H_2_O_2_, not PA, triggers the nuclear translocation of GmMPK6 during salt stress.

## 4. Materials and Methods

### 4.1. Plant Material

*Glycine max* L. seeds were surface-sterilized with bleach solution (0.2% Chlorox) for 5 min, followed by five washes with sterilized distilled water. Seeds were placed on wet paper towels for germination in a growth chamber (25 °C, 60% humidity) for 7 days under dark conditions. Before chemical or 300 mM NaCl treatments, all seedlings were stabilized in B&D solution [27] for at least 4 h. After the treatments, seedling roots were immediately fixed in fixation buffer (50% (*v/v*) absolute ethanol, 5% (*v/v*) acetic acid, 3.7% (*v/v*) formalin and 35% (*v/v*) D.W.) for immunolocalization assay, or immediately frozen in liquid nitrogen, and pulverized using mortars and pestles for protein blot analysis or GmMPK6 detection in the nuclei. Ground samples were stored as powder at −80 °C.

### 4.2. Subcellular Immunoblot Analysis

Subcellular fractionations were isolated using a rapid plant fractionation kit (Invent Biotechnologies, Plymouth, MA, USA). Briefly, the 7-day-old soybean seedlings were ground with buffer A; then, the sample was centrifugated at 1500× *g* for 5 min. For cytosolic fractions, the supernatant was transfer to a new tube and centrifuged at 4000× *g* for 10 min. The supernatant was transferred a new tube and centrifuged at 16,000× *g* for 30 min. The cytosolic fraction was obtained from the supernatant. For the nuclear fraction, the pellet of ground seedling was suspended with cold phosphate-buffered saline (PBS) and centrifuged at 1200× *g* for 5 min. The pellet resuspended with buffer B was centrifuged at 1200× *g* for 5 min, and the pellet resuspended with cold PBS was overlaid with buffer C followed by a centrifugation at 1200× *g* for 5 min. The nuclear fraction was obtained from the well-washed pellet. For immunoblotting, anti-GmMPK6 (Ab frontier, Seoul, Korea), anti-HIS (Abcam, Waltham, MA, USA), anti-ACT (Agrisera, Vännäs, Sweden), and HRP-conjugated secondary antibody (Abcam) were used. The signals were detected using a Fusion SL (Vilber Lourmat, France). All experiments were performed in triplicates, unless otherwise specified, with consistent results. Representative image data are shown.

### 4.3. Immunolocalization Assay

Paraffin-embedded roots were cut and fixed onto a slide glass, and the paraffin was removed by xylene. The samples were treated using ethanol for rehydration and washed with phosphate buffered saline Tween 20 (PBST; 137 mM NaCl, 1.5 mM KH_2_PO_4_, 2.7 mM KCl, 8 mM Na_2_HPO_4_, and 0.5 mL Tween 20). The slides were incubated in blocking buffer (5% (*w/v*) non-fat milk powder in PBST) for 45 min and washed using PBST. After the slides were treated with the anti-GmMPK6 antibody, washed 3 times using PBST, a FITC-conjugated antibody was added. The slides were washed, and the signal was measured using the z-stack method of a confocal microscope (LSM510, Carl Zeiss); 0.25 μg/mL of 4′,6-diamidino-2-phenylindole (DAPI) was used to detect the nucleus.

### 4.4. Nuclei Isolation and GmMPK6 Detection

Nuclei were isolated from ground soybean samples by PARTEC nuclei extraction buffer (Münster, Germany). Briefly, 400 μg of ground soybean root tissue was poured into 200 μL of the nuclei extraction solution and incubated on ice for 20 min and filtered through a 30 μm mesh. The filtrate was centrifuged at 10,000× *g* for 15 min and the pellet was resuspended by 200 μL PARTEC staining buffer. Anti-GmMPK6 antibody and FITC-conjugated secondary antibody were subsequently treated to the solution and washed by the staining buffer. The GmMPK6 signal was detected with confocal microscopy (LSM510, Carl Zeiss) and the intensity was measured by the Image J program and calculated using the following equation:

$$Signal\;intensity=\frac{Total\;intensity}{Number\;of\;Nuclei}$$

then, the analyzed relative signal intensity was compared with the control.

## Data Availability

The original data are provided in the manuscript.
